# Spatial association of racial/ethnic disparities between late-stage diagnosis and mortality for female breast cancer: where to intervene?

**DOI:** 10.1186/1476-072X-10-24

**Published:** 2011-04-04

**Authors:** Nancy Tian, J Gaines Wilson, F Benjamin Zhan

**Affiliations:** 1 Texas Center for Geographic Information Science, Department of Geography, Texas State University-San Marcos, 601 University Drive, San Marcos, Texas, 78666, USA; 2 Department of Chemistry and Environmental Sciences, University of Texas at Brownsville, 80 Fort Brown - MO1.114, Brownsville, Texas, 78520, USA; 3 Texas Center for Geographic Information Science, Department of Geography, Texas State University-San Marcos: 601 University Drive, San Marcos, Texas, 78666, USA; 4 School of Resource and Environmental Science, Wuhan University, Wuhan, 430079, PR China

## Abstract

**Background:**

Over the past twenty years, racial/ethnic disparities between late-stage diagnoses and mortality outcomes have widened due to disproportionate medical benefits that different racial/ethnic groups have received. Few studies to date have examined the spatial relationships of racial/ethnic disparities between breast cancer late-stage diagnosis and mortality as well as the impact of socioeconomic status (SES) on these two disparities at finer geographic scales.

**Methods:**

Three methods were implemented to assess the spatial relationship between racial/ethnic disparities of breast cancer late-stage diagnosis and morality. First, this study used rate difference measure to test for racial/ethnic disparities in both late-stage diagnosis and mortality of female breast cancer in Texas during 1995-2005. Second, we used linear and logistic regression models to determine if there was a correlation between these two racial/ethnic disparities at the census tract level. Third, a geographically-weighted regression analysis was performed to evaluate if this correlation occurred after weighting for local neighbors.

**Results:**

The spatial association of racial disparities was found to be significant between late-stage diagnosis and breast cancer mortality with odds ratios of 33.76 (CI: 23.96-47.57) for African Americans and 30.39 (CI: 22.09-41.82) for Hispanics. After adjusting for a SES cofounder, logistic regression models revealed a reduced, although still highly significant, odds ratio of 18.39 (CI: 12.79-26.44) for African-American women and 11.64 (CI: 8.29-16.34) for Hispanic women. Results of the logistic regression analysis indicated that census tracts with low and middle SES were more likely to show significant racial disparities of breast cancer late-stage diagnosis and mortality rates. However, values of local correlation coefficients suggested that the association of these two types of racial/ethnic disparities varied across geographic regions.

**Conclusions:**

This study may have health-policy implications that can help early detection of breast cancer among disadvantaged minority groups through implementing effective intervention programs in targeted regions.

## Background

The notable declines in late-stage diagnosis and mortality rates for breast cancer over the past twenty years have largely been attributed to improvements in early interventions among the population in general [1,2]. However, over this same period, racial/ethnic disparities between late-stage diagnoses and mortality outcomes associated with breast cancer have widened due to disproportionate medical benefits that different racial/ethnic groups have received [3,4]. It is well recognized that African-American and Hispanic women are more likely to be diagnosed in the advance stages of breast cancer, a fact that has been attributed to the lower survival rates and higher mortality rates in the two groups [5]. According to Surveillance Epidemiology and End Results (SEER) data collected between 1995 and 2001, 42% of African-Americans and Hispanics overall were not diagnosed until the later stage for all breast cancer cases, compared to 33% of White women for the same measure [4]. Mortality rates were observed to be 50% higher for African-American women than non-Hispanic White women, while Hispanic women had the lowest relative mortality [6]. A similar study that utilized SEER data from 1992 to 2000 performed a comparison of adjusted mortality rates relative to non-Hispanic Whites and found an increased risk for Hispanic Whites with a relative risk of 1.22 (95% Confidence Interval (CI): 1.16-1.28) and for African-Americans with a relative risk of 1.75 (CI: 1.68-1.82) [7].

Causes and mechanisms of racial/ethnic disparities remain unclear in breast cancer [8]. The higher breast cancer mortality among African-American women results in part from the poor survival rates, which is highly associated with stage at diagnosis [5]. Thus, racial/ethnic disparities in late-stage diagnosis propagate their effects on existing racial/ethnic disparities in breast cancer mortality. Differences in stage at diagnosis depend on socioeconomic status to a great extent, which plays an important role in the quality and quantity of cancer treatments [9]. Both late-stage diagnosis and socioeconomic status place direct impacts on racial/ethnic disparities in breast cancer mortality. Cancer cases can be categorized based on how far the carcinoma spreads from an original point [10]. A localized cancer is constrained to the organ of the breast and has not spread further than the breast tissue; regional cancer extends beyond the limits of the breast organ; and distant stage cancer occurs when tumor cells have metastasized to other parts of the body[11].

Few breast cancer studies have directly examined how racial/ethnic disparities at late-stage diagnosis and mortality vary across geographic regions, although some studies have been conducted to examine the geographic difference of stage at diagnosis and mortality [12,13]. A study of state-wide SEER data by stage has shown that Wyoming has the lowest rate of localized stage (56.5% of all diagnosed cases for breast cancer) and highest rate of distant (5.8%) breast cancer, with Alaska having the highest rate of regional breast cancer (33.2%) [7]. Among White women at age 50-64, the relative risk for breast cancer mortality decreased from 1.48 in 1950-1959 to 1.15 in 1990-1999 in the Northeast compared to that in the South, and African-American women experienced a downward trend of relative risk for the same age group from 1.13 in 1970-1979 to 1.00 in 1990-1999 [14]. This finding implies that White women experienced a larger proportion of decline in mortality than African-American women among the same age group.

Disparities between breast cancer late-stage diagnosis and mortality have not been compared at the census tract level across a large geographical area. Based on previous work by the authors [15], it was found that, in analysis results of racial/ethnic disparities at larger spatial scales (zip code and county level) relative to finer scales (e.g. census tract), there may be an obfuscation effect on racial/ethnic disparities across geographic regions due to aggregation. The study presented in this article, in contrast, intends to determine if there is any spatial relationship in racial/ethnic disparities between late-stage diagnosis and mortality for breast cancer as well as how socioeconomic status (SES) impacts the number of census tracts that are significant in both late-stage diagnosis and mortality and the spatial relationship between these two types of racial/ethnic disparities. An investigation of racial/ethnic disparities across different geographic regions may provide useful insights revealing the effects of unknown risk factors for late-stage diagnosis and higher breast cancer mortality among minority groups and facilitate the allocation of cancer prevention education and health care resources more effectively and efficiently. Investigation of SES influences on racial/ethnic disparities could provide some insights on the interaction between stage diagnosis and the continuous influence on survival rates of breast cancer. There may also be an opportunity to understand how socioeconomic status plays a role in determining cancer stage at diagnosis and mortality, especially in impoverished regions.

## Methods

### Data Description

We obtained individual breast cancer incidence and mortality cases from 1995 to 2005 from the Texas Cancer Registry and the Vital Statistic Unit, Texas Department of State Health Services [6]. Late-stage incidence and mortality of breast cancer were computed by adjusting to the 2000 U.S. standard million population for each census tract in Texas for non-Hispanic Whites, African-Americans and Hispanics. According to SEER Summary Stage Coding Scheme [10], this study defined regional and distant (i.e. tumor cells metastasizing to other body parts) stages as late-stage breast cancer [16,17].

In all, 44,515 cases were identified as late-stage over the period 1995-2005, which accounted for 29.75% of total breast cancer cases. Moreover, African-American and Hispanic women had a 10% greater proportion of late-stage diagnosis for breast cancer compared to their non-Hispanic White counterparts. The three racial/ethnic groups had a total of 26,910 mortality cases reported in Texas from 1995-2005. All but 3,228 (about 12%) of mortality cases in this study could not be geocoded, largely due to incomplete address information.

In order to assess the relationship between SES and racial/ethnic disparities of late-stage diagnosis and mortality for breast cancer, the percentage of population under the poverty level in each census tract was used as a surrogate of SES, as poverty level is significantly correlated with other measures including median income, education level and unemployment rate [18]. Census tracts were classified into low, middle and high SES groups based on <10%, 10% ~20% and >20% population living below the poverty level. This same coding scheme is used in SEER reporting by the National Cancer Institute (NCI) [18].

### Statistical Methodologies

Three methods were implemented to assess the spatial relationship between the racial/ethnic disparities of breast cancer late-stage diagnosis and mortality. First, we examined the number and locations of census tracts that tested significant in two types of racial/ethnic disparities. Second, we used bivariate linear and logistic regression models to determine if there was a correlation between these two racial/ethnic disparities at the census tract level. Third, a geographically-weighted regression analysis was applied to evaluate if this correlation occurred after weighting for local neighbors.

#### Significance tests of racial/ethnic disparities

This study utilized an absolute measure which is the arithmetic rate difference (RD) between the target group (minority) and the reference group (non-Hispanic Whites). Because relative (e.g. rate ratio) measures rely heavily upon the baseline, absolute measures of cancer disparities are preferred to reflect the actual difference for policy-makers, who can help take actions on allocating limited health resources [19]. Thus, the inconsistencies on disparity measures of the same health outcomes may occur when using absolute and relative tools due to the inherent qualities of these two different measures. However, only a few census tracts tested significant using a relative measure, which would create difficulties in establishing a regression model to assess spatial relationships of racial/ethnic disparities between late-stage diagnosis and mortality.

Compared to non-Hispanic Whites, absolute statistics of racial/ethnic disparities for late-stage incidence and breast cancer mortality were estimated using a population-weighting scheme in each census tract for African-American and Hispanic women. Two-tailed significance tests were employed with *p *value of 0.05 by comparing the RD statistic to its expected distribution under the null hypothesis: equal rates for the target and reference groups. Multiple testing adjustments were implemented to correct the inflation of type I error due to multiple individual tests for geographic datasets. The details about the significance test can be found elsewhere [20].

#### Bivariate linear and logistic regression analyses

We used the disparity statistics derived from significance tests of racial/ethnic disparities adjusting for population sizes in linear regression models to assess whether census tracts with high racial/ethnic disparities of late-stage diagnosis in breast cancer had high racial/ethnic disparities of breast cancer mortality and vice versa. Due to some outliers and extreme values in the data, disparity statistics were transformed to conform to a normal distribution. The normal-transformation was performed by ranking values of disparity statistics from the lowest to the highest and assigning the values of normal distribution according to their ranks [21]. The regression parameters were estimated using a maximum likelihood approach [22] and the F distribution was adopted to evaluate the significance of the overall model [23].

Logistic regression models were constructed based on the outcome of significance tests between racial/ethnic disparities of late-stage diagnosis and mortality in the census tract level. Both dependent and independent variables had binary values of 0 (not significant) and 1 (significant) for each census tract. The parameter estimation is a repetitive process with the goal of maximizing the log-likelihood. The significance of the full model and individual parameters were assessed using a Chi-square distribution [24]. The regression analyses were conducted in STIS 1.8, developed by BioMedware [25].

#### Geographically-weighted regression (GWR) analyses

Spatial autocorrelation was tested for and found within the residuals from linear regressions using Local Moran's *I *[26]. This test indicates whether racial/ethnic disparities between late-stage diagnosis and mortality rates displayed a greater likelihood with similar values in adjacent census tracts than among census tracts further away. Spatial lag and spatial error models were not utilized in this study because these two methods are not able to estimate the local correlation coefficients for each census tract [27]. To evaluate the strength of the correlation of racial/ethnic disparities between late-stage diagnosis and mortality accounting for the local geographical weight around a focal point, geographically-weighted regression models were constructed to weigh the neighborhoods using ArcInfo (Version 9.3) [28,29]. However, logistic regression models with geographical weighting could not converge to estimate model coefficients using the maximum likelihood approach. Thus, only geographically-weighted linear regression was conducted in this study.

The weighting scheme utilized in this study was the Gaussian kernel function [30]. The Gaussian kernel bandwidth varies across space with the decay function of weighting neighborhoods. The kernel type was set up as adaptive to account for the density of spatial features and the optimal bandwidth was determined by minimizing Akaike Information Criterion (AICc) [31]. The parameter estimates were mapped in ArcInfo (Version 9.3).

## Results

### Spatial locations of significant racial/ethnic disparities

Table 1 shows the number of census tracts that tested significant in the two racial/ethnic disparities between breast cancer late-stage diagnosis and mortality using the RD measure. These census tracts were further sorted based on low, middle and high SES measured by percentage of people living under the poverty line. For both African-Americans and Hispanics, more census tracts were identified with statistically significant racial/ethnic disparities in breast cancer mortality than in late-stage diagnosis. It was observed that about 188 census tracts had African-American women with a significantly higher late-stage diagnosis rate and 278 census tracts with a significantly higher breast cancer mortality rate relative to their non-Hispanic White counterparts. Furthermore, 79% and 68% of these census tracts, respectively, had low SES for breast cancer late-stage diagnosis and mortality among African-Americans. A similar pattern was observed for Hispanics who experienced significantly higher rates of breast cancer late-stage diagnosis and mortality in 266 and 328 census tracts, respectively. Among Hispanic women, 88% and 81% census tracts that tested significant in racial/ethnic disparities of breast cancer late-stage diagnosis and mortality, respectively, had low SES with more than 20% population living the poverty line.

**Table 1:** Number of census tracts with significant racial disparities in breast cancer late-stage diagnosis and mortality for African-American and Hispanic women using the RD measure

			**High SES**^**1**^	**Middle SES**^**1**^	**Low SES**^**1**^	Total (N)
African- Americans	Late-stage Diagnosis	RD >0	13	26	149	188
	Mortality	RD >0	34	54	190	278

Hispanics	Late-stage Diagnosis	RD >0	8	23	235	266
	Mortality	RD >0	20	42	266	328

^1^SES (Socioeconomic status) is categorized as follows: high SES (0-9.99% population under the poverty line), middle SES (10.00-19.99%), low SES (20.00%).

**Figure 1:**
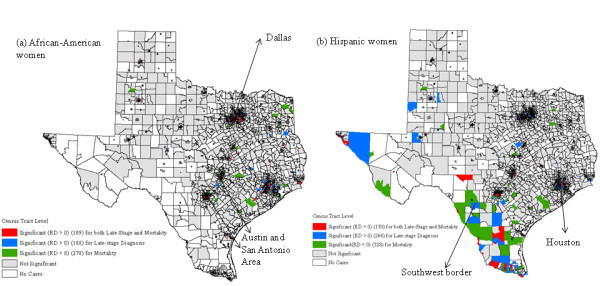
**Geographic distributions of census tracts with significant racial disparities in late-stage diagnosis and mortality for breast cancer using the RD measure for both African-American (a) and Hispanic women (b)**.

However, the census tracts that tested significant for racial/ethnic disparities both in breast cancer late-stage diagnosis and mortality did not fully overlap across space (Figure 1). Among African-Americans, 109 census tracts tested significant in racial/ethnic disparities for both breast cancer late-stage diagnosis (188) and mortality (278) (Figure 1a). These significant census tracts were primarily found within the three metropolitan areas of Houston, Dallas and Austin-San Antonio. Among Hispanics, 130 census tracts tested significant in racial/ethnic disparities for both breast cancer late-stage diagnosis (266) and mortality (328) (Figure 1b). These tracts were found within these three metropolitan areas as well as on the Southwest U.S.-Mexico border in Texas where the Hispanic population was greater. The downtown regions of these areas displayed significant elevated late-stage diagnosis and mortality rates of breast cancer for African-Americans and Hispanics compared to non-Hispanic Whites.

### Correlations

Table 2 shows the results of linear and logistic regression models and Moran's *I *test on the residuals. First, all regression models were significant with the *p *values less than 0.001, which implied that racial/ethnic disparities in late-stage diagnosis was a legitimate predictor to test if a census tract was significant in racial/ethnic disparities of breast cancer mortality. The correlation coefficients were 0.23 in the linear regression models with normalized RD statistics of late-stage diagnosis as the independent variable and breast cancer mortality as the dependent variable for African-American and Hispanic women. Spatial autocorrelation tested significant within the residuals of the linear regression based on the Moran's *I *and the minimum *p *value of 0.001 indicated the existence of spatial autocorrelation within the regression models. The Moran's *I *index suggested that a slight similarity in disparities was detected for neighbor census tracts.

**Table 2:** Linear and logistic regression results and Moran's *I *test on residuals with the dependent variable of racial disparities in breast cancer mortality and independent variable of racial disparities in late-stage diagnosis for African-American and Hispanic women.

	Regression Type	Coefficient/Odds Ratio	*p *(Regression)	Moran's I	*p *(Moran's I)
African-Americans	Linear	0.23	<0.001	0.04	0.001
	Logistic	33.76 (CI: 23.96-47.57)	<0.000001	0.08	0.001
		18.9 (CI: 12.79-26.44) (SES*-adjusted)	<0.000001	0.062	0.001

Hispanics	Linear	0.23	<0.001	0.04	0.001
	Logistic	30.39 (CI: 22.09-41.82)	<0.000001	0.14	0.001
		11.64 (CI: 8.29-16.34) (SES*-adjusted)	<0.000001	0.086	0.001

*In the logistic regression models, SES (Socioeconomic status) is categorized as follows: high SES (0-9.99%), middle SES (10.00-19.99%), low SES (20.00%).

Table 2 also summarizes the results of logistic regression testing the spatial relationship of the two racial/ethnic disparities using RD measure. The full models and individual model parameters tested significant with the *p *value less than 1.0 E^-5^. The much higher odds ratio reflected the strong spatial connection of racial/ethnic disparities between breast cancer late-stage diagnosis and mortality. For example, if a census tract was identified to have significant racial/ethnic disparities in late-stage diagnosis, the census tracts were 33.76 (CI: 23.96-47.57) times more likely to display significant higher mortality rates for African-Americans and 30.39 (CI: 22.09-41.82) times for Hispanics. After adjusting for the SES cofounder, logistic regression models found a reduced, although still highly significant, odds ratio of 18.39 (CI: 12.79-26.44) for African-American women and 11.64 (CI: 8.29-16.34) for Hispanic women. In addition, spatial autocorrelations were tested within the residuals of logistic regression models constructed for both African-American and Hispanic women and found to be significant.

**Figure 2:**
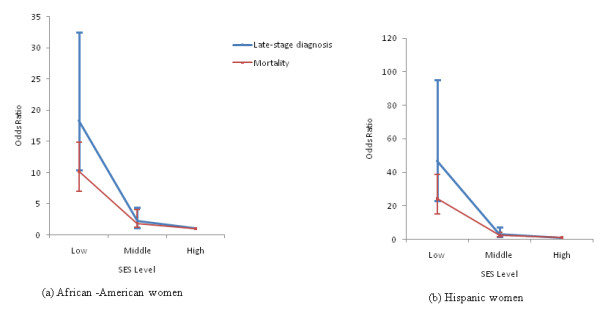
**The odds ratios (95% CI) of SES level on racial disparities in breast cancer late-stage diagnosis and mortality for African-American (a) and Hispanic women (b)**.

The relationships between racial/ethnic disparities and SES levels were further assessed in logistic regression models for both breast cancer late-stage diagnosis and mortality. Figure 2 displays the results of the logistic regression with odds ratio of the parameters and significance of the model. The relationship between the two racial/ethnic disparities and SES levels tested significant (*p *< 0.05) in all models. For African-American women (Figure 2a), census tracts classified as middle and low SES were 2.27 (CI: 1.13-4.40) and 18.35 (CI: 10.35-32.52) times more likely to report significant racial/ethnic disparities in late-stage diagnosis, while the odds ratios were 1.86 (CI: 1.20-2.89) and 10.19 (CI: 7.00-14.82) for breast cancer mortality. Within census tracts of middle and low SES level, Hispanics were found to be 3.24 (CI: 1.45-7.27) and 46.89 (CI: 23.07-95.30) times more likely to be associated with significantly higher rates of late-stage diagnosis than non-Hispanic White females. These ratios were 2.51 (CI: 1.46-4.29) and 24.29 (CI: 15.30-38.57) for breast cancer mortality (Figure 2b). Furthermore, the impacts of SES level on racial/ethnic disparities were greater for late-stage diagnosis than for mortality as well as for Hispanic women than for African-Americans. There was also a marked difference by SES when comparing odds ratio disparities between late-stage and mortality for each of the race/ethnicities. In the poorest census tracts, the odds ratios were substantially higher for both African Americans and Hispanics, versus the middle and high SES tracts, where odds ratios were similar.

**Figure 3:**
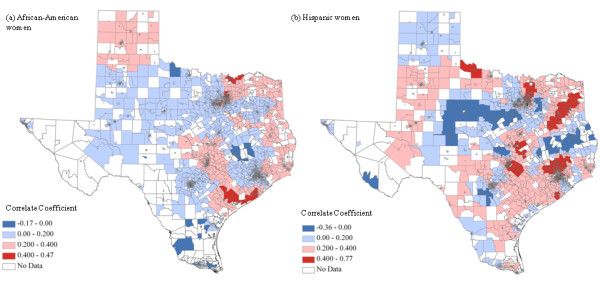
**Standardized correlation coefficients for racial disparities between late-stage diagnosis and mortality for breast cancer based on the GWR analysis using RD measure for African-American (a) and Hispanic women (b)**.

The correlation coefficient varied across space based on the linear geographically-weighted regression (GWR) model by taking into account the surrounding census tracts. African-American women had 96.8% of the census tracts with positive local correlation coefficients (Figure 3a) and Hispanic women had 94.6% (Figure 3b), which suggests a positive association of racial/ethnic disparities between breast cancer late-stage diagnosis and mortality. The correlation coefficient values fluctuated from 0.0002 to 0.44 for African-Americans and from 0.00002 to 0.77 for Hispanic women.

A positive relationship was observed for racial/ethnic disparities between breast cancer late-stage diagnosis and mortality. Further, the stronger correlation coefficients were found in the Southeast for both African-American and Hispanic women in Texas (Figure 3). For African-American women, the correlation coefficients ranged from 0.2-0.4 within the census tracts in the Austin area; Houston downtown had coefficients in the range of 0-0.2; and Dallas metropolitan area resulted in correlation coefficients from 0 to 0.47 (Figure 3a). For Hispanic women, the metropolitan areas of Austin and Dallas were observed with all four groups of correlation coefficients and Houston had positive correlation coefficients ranging from 0-0.77 (Figure 3b). Moran's *I *test, an essential spatial factor not included in many regression models, indicated the existence of spatial dependency within the residuals of bother linear and logistic regression models with the *p *value of 0.001 (Table 2). Although the adjusted regression models can help reduce the spatial dependency effects on disparity relationship of late-stage diagnosis and breast cancer mortality, the geographically-weighted logistic regression failed to converge for both African-American and Hispanic women.

## Discussion

The results of this study indicate that the spatial relationships of racial/ethnic disparities between breast cancer late-stage diagnosis and mortality were complex and non-stationary across Texas from 1995-2005. The dichotomous results from the significance test indicate that about 40% (109/278 or 130/328) of the census tracts with significant racial/ethnic disparities in breast cancer mortality also displayed significant disparities in late-stage diagnosis for both African-Americans and Hispanics, respectively. On the other hand, 58% of the census tracts tested significant in racial disparities of late-stage diagnosis displayed significant in breast cancer mortality as well for African-Americans, and the similar result (49%) were observed for Hispanics.

More census tracts were identified as having significant mortality disparities compared to census tracts having late-stage disparities. This implies that some other factors impact racial/ethnic disparities in breast cancer mortality. While stage of breast cancer at diagnosis is a critical factor in determining breast cancer survival, other factors such as treatment options and financial support are also crucial in ultimately saving women's lives [32,33]. Linking Detroit SEER registry with Medicaid enrollment data in Michigan, Bradley et al. in 2002 revealed that African-American women had less likelihood to undergo cancer surgery and radiation that contributed to the elevated mortality risk. In this study, there were more census tracts that tested significant in both late-stage and mortality disparities for Hispanics than for African-Americans due to the much higher Hispanic population resided in Texas [34].

Using regression analysis, this study provides additional evidence, in the form of a spatial perspective, of the strong and positive relationship between late-stage diagnosis and mortality of breast cancer with regard to racial/ethnic disparities. If a census tract tested significant in racial/ethnic disparities of late-stage diagnosis, this tract is more than 30 times more likely to be significant in racial/ethnic disparities of breast cancer mortality. Similarly, using Surveillance Epidemiology and End Results (SEER) data, Li and colleagues (2003) found that African-Americans were 2.3 and 2.5 times more likely to have the elevated risk of advance stage of breast cancer compared to non-Hispanic Whites. On the other hand, Hispanic women tested 1.8-fold in diagnoses at the late-stage of breast cancer. Furthermore, African-Americans and Hispanics were found to have 1.5 and 1.1 greater in mortality risk than Whites, even after adjusting age, SEER registry, stage, ER and PR status, surgical treatment and radiation therapy [5].

A large number of census tracts were found to have significantly higher breast cancer late-stage diagnosis and mortality rates for African-American and Hispanic women in comparison with non-Hispanic White females. This result strongly agrees with the argument that minority women tend to be diagnosed in the advanced stage of breast cancer relative to non-Hispanic White women. Richardson and colleagues [35] found that African-Americans and Hispanics were at 1.29- and 1.32-fold greater risk to be diagnosed in the advance stage of breast cancer in Los Angeles, California than White non-Hispanics, when SES was controlled. Hispanics are often reported to have lower incidence rates than non-Hispanic Whites [6]. However, the incidence rate ratio for Hispanics has been observed to be significantly lower in the early detection of breast cancer relative to non-Hispanic Whites, especially within the age group under 50 [36].

Census tracts that had significantly elevated mortality rates were associated with lower SES characteristics, mostly situated within the three metropolitan areas and the Southwest border with Mexico. This may be due to the fact that medical services, including access to health care, sufficient physicians and cancer treatment are more likely to be underserved in deprived neighborhood communities [32,37]. Lower socioeconomic status is an important determinant for the advanced stage at diagnosis and cancer treatment follow-ups that lead to lower survival and higher mortality rates, especially among susceptible minority groups [38].

Results of this study reveal that SES considerably interrelates with racial/ethnic disparities between stage diagnosis and mortality of breast cancer. Racial/ethnic disparities are widening, in part due to the greater decrease of advanced stage diagnosis and corresponding increase of survival rates for non-Hispanic White women compared to disadvantaged minority groups within same regions [39]. Women with lower socioeconomic status (SES) are more likely to be diagnosed at late-stage, which therefore may lead to higher mortality for these groups [40]. After adjusting SES cofounders, the odds ratio of disparities in late-stage diagnosis and breast cancer mortality were reduced dramatically with a tighter confidence interval. However, stage of diagnosis is still a critical factor in determining if a census tract is significant in racial/ethnic disparities of breast cancer mortality. The underlying factors including health care access, mammography screening and education etc., warrant the further investigation in order to help policy makers to allocate health-related resources with the ultimate purpose of reducing disparities in late-stage diagnosis and breast cancer mortality. Nevertheless, SES at aggregate levels may place some limitations on this study in that this contextual measure may not reflect the accurate SES for different racial/ethnic groups at the same geographic area because SES was measured by all people under the poverty level instead of each racial/ethnic group separately [41].

Compared to non-Hispanic Whites, African-American and Hispanic women are subject to elevated mortality rates in breast cancer. This disparity can be partially explained by the prevalence of late-stage diagnosis for minority breast cancer patients. Lannin et al. [42] found that African-American women with low-income had 3.0 higher odds of being diagnosed in late-stages. In addition, lack of private health insurance, delay in seeing a physician, lack of access to transportation, less utilization of mammography screening, and cultural beliefs may be important social barriers to preventing minorities from early diagnosis of breast cancer [43,44]. Despite lower breast cancer incidence for Hispanics, early stage diagnosis is less likely found among these women than non-Hispanic Whites [45].

The correlations between the racial/ethnic disparities of breast cancer late-stage diagnosis and mortality were shown to vary by region. More than 90% of census tracts were identified as having a positive association between the two racial/ethnic disparities. The strength on the variability in the association between the two racial/ethnic disparities may indicate different underlying risk factors impacting the stage diagnosis and mortality in breast cancer across space. For some regions that had a stronger relationship between the two racial/ethnic disparities, early intervention programs, such as mammography screening, may help to reduce late-stage diagnoses, in effect attenuating racial/ethnic disparities in mortality. For regions that have a weaker relationship, identifying other risk factors beyond stage diagnosis might be critical in closing racial/ethnic disparities in breast cancer mortality.

## Conclusions

The results reported here indicated a 40% overlap in census tracts that tested significant for both racial/ethnic disparities of breast cancer late-stage diagnosis and mortality. The socioeconomic level for each census tract is a fundamental determinant in the outcomes of the significance tests of both types of disparities in breast cancer outcomes. Based on the results of a regression analysis using data at the census tract level, this study found that there existed a positive spatial association of these racial/ethnic disparities for both African-American and Hispanic women. The regions with a weaker relationship between the two racial/ethnic disparities suggest that further investigation is warranted to understand underlying risk factors including cancer treatment and cultural factors, which may be responsible for these specific results between stage diagnosis and breast cancer mortality. The discrepancies in racial/ethnic disparities reported in this paper imply that different risk factors have an influence on racial/ethnic disparities between diagnosis at advance stages and mortality of breast cancer in different geographic regions. Future work should concentrate on identifying risk factors contributing to racial/ethnic disparities between late-stage diagnosis and mortality as well as better understanding how intervention programs would improve the rates of early detection and breast cancer survival for all women.

## Competing interests

The authors declare that they have no competing interests.

## Authors' contributions

NT designed and performed the statistical analysis as well as wrote the main body of the article. JGW significantly contributed the interpretation of the results and revised the article. FBZ helped the study design, interpreted the results, and participated in the preparation of the manuscript. All authors have read and approved the final version of the manuscript.
